# Impact of Primary Care Delay on Progression of Breast Cancer in a Black African Population: A Multicentered Survey

**DOI:** 10.1155/2019/2407138

**Published:** 2019-08-07

**Authors:** Olayide Agodirin, Samuel Olatoke, Ganiyu Rahman, Julius Olaogun, Oladapo Kolawole, John Agboola, Olalekan Olasehinde, Aba Katung, Omobolaji Ayandipo, Amarachukwu Etonyeaku, Anthony Ajiboye, Soliu Oguntola, Oluwafemi Fatudimu

**Affiliations:** ^1^Department of Surgery, University of Ilorin and University of Ilorin Teaching Hospital, Nigeria; ^2^Department of Surgery, Cape Coast Teaching Hospital, Cape Coast, Ghana; ^3^Department of Surgery, Ekiti State University Teaching Hospital, Ado-Ekiti, Nigeria; ^4^Department of Surgery, LAUTECH Teaching Hospital, Osogbo, Nigeria; ^5^Department of Surgery, Obafemi Awolowo Teaching Hospital, Ile-Ife, Nigeria; ^6^Department of Surgery, Federal Medical Center, Owo, Nigeria; ^7^Department of Surgery, University College Hospital, Ibadan, Nigeria; ^8^Department of Surgery, Obafemi Awolowo Teaching Hospital, Ilesha, Nigeria; ^9^Department of Surgery, Bowen University Teaching Hospital, Nigeria; ^10^Department of Surgery, LAUTECH Teaching Hospital, Ogbomoso, Nigeria; ^11^Department of Surgery, Federal Teaching Hospital, Ido-Ekiti, Nigeria

## Abstract

**Background:**

Reports are scanty on the impact of long primary care interval in breast cancer. Exploratory reports in Nigeria and other low-middle-income countries suggest detrimental impact. The primary aim was to describe the impact of long primary care interval on breast cancer progression, and the secondary aim was to describe the factors perceived by patients as the reason(s) for long intervals.

**Method:**

Questionnaire-based survey was used in 9 Nigerian tertiary institutions between May 2017 and July 2018. The study hypothesis was that the majority of patients stayed >30 days, and the majority experienced stage migration in primary care interval. Assessment of the impact of the length of interval on tumor stage was done by survival analysis technique, and clustering analysis was used to find subgroups of the patient journey.

**Results:**

A total of 237 patients presented to primary care personnel with tumor ≤5cm (mean 3.4±1.2cm). A total of 151 (69.3%, 95% CI 62.0-75.0) stayed >30 days in primary care interval. Risk of stage migration in primary care interval was 49.3% (95% CI 42.5%-56.3%). The most common reasons for long intervals were symptom misinformation and misdiagnosis. Clustering analysis showed 4 clusters of patients' experience and journey: long interval due to distance, long interval due to misinformation, long interval due to deliberate delaying, and not short interval—prepared for treatment.

**Conclusion:**

The majority of patients stayed longer than 30 days in primary care interval. Long primary care interval was associated with a higher risk of stage migration, and more patients reported misinformation and misdiagnosis as reasons for a long interval.

## 1. Background

The 2018 status report on the global burden of cancer showed that the incidence of breast cancer is fast catching up with that of lung cancer as the most common cancer in both sexes combined [1] with an estimated 2.1 million new cases in 2018. Also in the same report, breast cancer was the most commonly diagnosed cancer in more than 80% of countries in the world (154 of 185) [1] and the most common cause of cancer-related deaths. Low-middle-income countries (LMICs) suffer a disproportionately high rate of breast cancer (BC) mortality compared to their share of incidence because of late diagnosis and treatment.

Factors responsible for late diagnosis and treatment of breast cancer in LMICs may reside in one of 3 intervals described in the Danish model for cancer delays [2] and adopted in the Aarhus statement [3]: the patient interval, the doctor interval, or the system interval. The patient interval is the time between symptom detection and first physician contact; the doctor interval is the time between first physician contact and initiation of investigations; and the system interval is the time between investigation and the start of treatment. The continuum from first physician contact to the start of treatment has two subparts: the primary care and secondary care intervals [2]. Primary care interval is between first physician contact and referral, and secondary care interval is between referral and start of treatment. Researchers use the terminologies interchangeably, and they often refer to the intervals as delays [4]. The term interval is preferred [3, 4]. Henceforth, we will adopt the terms patient, primary care, and secondary care intervals. And system interval will be a collective term for primary and secondary care intervals combined.

The system interval is underreported worldwide, [5, 6] because the healthcare system is organized and usually efficient in high-income countries (HICs) and researchers in LMICs often blame long patient interval for most long intervals [5, 7]. Irrespective of the events prolonging the intervals, researchers agree that long patient interval and long total interval (detection to treatment interval) impact negatively on tumor progression, cost of treatment, and flexibility of surgical intervention. A long system interval should also be detrimental judging from the known association. Surprisingly, reports on aspects of system interval are conflicting [6, 8–11]. While some researchers concluded that long primary care interval is detrimental [10, 12, 13], others explicitly stated no association between some situations of extended system interval and outcome of breast cancer. Afzelius et al. in a study of over 7000 breast cancer patients reported that physician intervals longer than 15 days were associated with better prognosis, and they argued that physicians' ability to distinguish more aggressive from less aggressive tumors explained their results [14]. Another study by Sainsbury et al. found no evidence that system interval longer than 90 days adversely influenced survival [15]. Recent separate studies by Redaniel et al. 2013 [16] and Bleicher et al. 2016 [11], correlating surgery waiting time and survival, found that longer waiting time within 60 days of diagnosis did not significantly impact survival.

The literature on the impact of the various intervals on breast cancer progression in LMICs is scanty, and research on system interval is rare. Nonetheless, mounting evidence from secondary analyses suggests a growing contribution of events in the system interval to long intervals [17] and breast cancer progression or stage migration in LMICs [18]. Ayoade [19] et al. in Nigeria attributed 32% rise in the rate of late presentation to events in primary care. Also in Nigeria, Akinkolie et al. [20] reported a shorter time to specialist presentation among patients who presented directly to oncologist compared to those who presented after visiting a primary care provider, and Ezeome et al. [21] reported that a long primary care interval was present in 72.4% of breast cancer patients. In Ghana Clegg-lamptey et al. [22] reported that prior consultation with a physician was the most common cause of long interval, and in India Chintamani et al. [23] reported that the significant factors associated with extended time to diagnosis among breast cancer patients were system related.

The primary objective in this research was to describe the impact of the length of primary care interval on breast cancer progression among patients in Nigeria, and the secondary objective was to describe the factors perceived and reported by patients as the cause(s) of long interval.

## 2. Method

### 2.1. Design and Settings

This research was a cross-sectional questionnaire-based survey in 9 public tertiary health institutions in North Central and Southwestern Nigeria. At the time of the study, Nigeria had limited specialist coverage, and healthcare delivery was pluralistic. There was no functional protocol for referral of BC patient. Hence patients' preference directed their choice of medical personnel. The research institutions accepted self-referrals, referrals from individual health personnel, or referrals from public primary and secondary health hospitals and private hospitals.

### 2.2. Data Collection

After obtaining ethical clearance from the ethical review boards of the participating institutions and respondents, trained assistants administered the semistructured questionnaires to respondents in a face-to-face interview. Face and content validity of the questionnaire was conducted by the authors, and then it was pretested in a pilot study with 30 breast cancer patients. Information collected was respondent's sociodemography, recall of breast change, knowledge of breast cancer treatment, time to first orthodox contact, time to specialist contact, and tumor size estimate at detection, at contact with first healthcare provider (FHP) consulted, and at arrival in the specialist clinic.

Changes in estimated tumor size/stage along the continuum from first symptom detection to arrival in the specialist clinic constituted the primary variable. The other variables of interest included were the designation of the FHP and number of health personnel visited, distance to a specialist clinic, and reasons for the delay. The operational definitions of the intervals were adapted from the definition in previous researches [2, 3, 24]. Patient interval was the period from detection of first breast change to first orthodox medical consultation, primary care interval was the period from the first orthodox consultation to arrival in the specialist clinic, and symptom detection to the specialist clinic was the period from symptom detection to arrival in a specialist clinic. The intervals were in days, weeks, or months. To calculate intervals in days, recordings made in weeks were multiplied by 7 and recordings made in months were multiplied by 30. The interval was long if the patient interval was more than 60 days, if primary care interval was more than 30 days, and if symptom detection to the specialist interval was more than 90 days

The respondents were interviewed within four weeks of arrival in the specialist clinic to minimize recall bias, and they were helped to cast their mind back on significant personal, social, regional, or national events surrounding the recalled periods. The interview was in the patient's mother tongue or English as preferred by the respondent.

The interviewers administered the questionnaire as a schedule. The specific questions regarding the lump detection and size included the following: what drew attention to the lump; how long ago was the lump noticed; please estimate the size of the lump using digit or fist. The specific questions regarding personnel consultation included the following: which orthodox personnel was first visited to receive treatment; what the directive of the FHP on investigation or treatment was; why there was delay in presenting here (specialist clinic). Specific questions regarding awareness included the following: awareness of breast cancer before noticing the first symptom; awareness of the treatment for breast cancer; and awareness of someone treated for breast cancer.

Tumor size (T) estimate was the surrogate for disease stage using the T1-3 as in the American Joint Committee on Cancer staging for breast cancer. The patients estimated the perceived tumor size by using their phalanx, finger, or clenched fist(s). The size shown was then estimated on a ruler marked in centimeters. The patients were required to estimate size at detection, at contact with FHP, and at arrival in the specialist clinic. The size of tumor estimated at arrival in the specialist clinic was the current tumor size (passive eliminated). The patients' records were considered unreliable and excluded if current size perceived was more than 2cm different from the size measured physically and recorded in the case note by the attending clinician in the specialist clinic.

### 2.3. Sampling

Based on the report in Nigeria that about 72.4% of BC patients delayed for longer than three months between first physician contact and initiation of treatment [21]. The hypothesis was that the majority of patients stayed >30 days and the majority experienced stage migration in primary care interval. The required sample size was 215 respondents who arrived at FHP with early-stage disease (T ≤5cm (T1 & T2 size)) based on the sample size calculation for descriptive cross-sectional study at absolute precision of 5% and 90% (1.64) confidence level. In anticipation of 10% nonresponse, the sample size was increased to 237. Sampling was purposive convenience method between May 2017 and July 2018. Recruitment included only newly diagnosed consecutive consenting female patients. Mental incapacitation and the barrier in language were exclusion criteria. Patients who could not estimate their tumor size were excluded. Patients who had bilateral tumors were captured once based on the first side noticed. Exclusion criteria included the patients who perceived tumor size >5cm (T3 size) at arrival at the first medical caregiver because they already attained the maximum tumor size based on the staging method. Hence disease progression could no longer be assessed based on the change in tumor size only.

### 2.4. Statistical Analysis

The collected information was coded and transferred into specially designed 2016 Microsoft Access database using Microsoft Access form. Statistical analysis was carried out by EasyR, freely available statistical software, and SPSS version 20.

Change in tumor size between the contact with FHP and the arrival in a specialist clinic was compared using paired t-test. Stage migration was analyzed using the survival analysis technique. The risk (probability) of stage migration was within confidence limits. Exploratory multivariate partitioning to find patient clusters associated with length of primary care interval was done by cluster analysis. For the cluster analysis, the hierarchical agglomerative method [which does not require prespecifying the number of subgroups] was first used to explore the data, and then the clusters were optimized using the K-means partitioning after sorting in ascending order of unique numbers. The variables used for clustering were dichotomized dummy coded values of the following: provider interval (≤30 days (0)/>30 days (1)), FHP directive (correct (0)/incorrect (1)), tumor size at FHP contact (≤3cm (1)/>3cm (0)), number of orthodox personnel visited (1 (0) />1 (1)), driving distance to specialist ( ≤60 minutes (0) and below/>60min (1)), and age (≤40 years (1) / >40 years (0)). Descriptive statistics were by mean, median, and quartiles. The reasons for the long intervals were in a frequency table. P-value for inferential statistic was set at 5%.

## 3. Results

A total of 237 respondents were eligible with tumor size estimated ≤5cm after approaching 427 respondents. This report presents the results of the analyzed 237 responses. Among the 190 excluded records, 12 could not describe size both at the time of lump detection and at arrival in the FHP, 8 could not describe the size at the FHP alone, and 170 tumors already exceeded 5cm at arrival at the FHP [arrival at FHP with T1 or T2 disease was 56% (95% CI 50.6-60.3)]. No respondent was excluded because of mental incapacitation or language barriers. A total of 76 (32.0%) respondents were recruited in North Central and 161 (68%) in Southwestern Nigeria. The modal age range was 41 and 50 years. The majority were married, and the majority had at least a secondary education. The sociodemographics are as shown in Table 1. Total of 171 respondents (77.0%, 95%CI 71.0-82.0) were aware of breast cancer before detecting their first symptom. Breast lump was the most frequently reported first symptom (Table 1).

The majority of patients stayed longer than 30 days in the primary care interval (n=151, 69.3%, 95% CI 62.0-75.0) and only a third of respondents arrived in the specialist clinics within 30 days of detecting their first symptom (n=76, 33.6%, 95%CI 27.5-40.0). The growth in tumor size during the primary care interval (2.8cm±4.2cm) was significantly greater than that in the patient interval (0.6cm±1.0) (p=0.0001, paired t-test) (Table 2).

**Figure 1:**
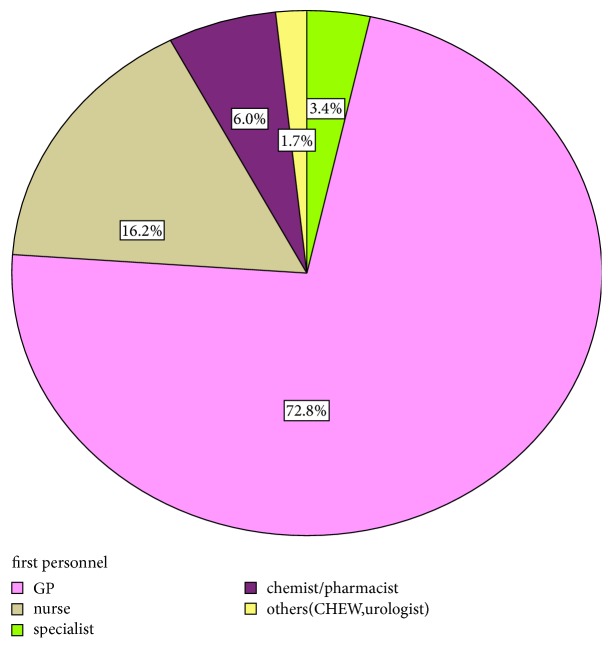
First healthcare provider (FHP) consulted: two-thirds of patients first visited a doctor who was a general practitioner.

Using the survival analysis technique, the risk of migration in primary care interval 49.3% (95% CI 42.5%-56.3%) was higher than the risk of migration in the patient interval 20.0%, (95% CI 15%-25.8%) [the risk difference was 29.3% (95% CI 20.8-37.8), and the odds ratio (OR) was 2.3 (95% CI 1.7-3.1)]. The risk of stage migration increased as the patient stayed longer in the primary care interval. The odds ratio of stage migration for patients who stayed 31 to 90 days in primary care interval was 3.0 (95%CI 1.0-8.5) compared to 20.2 (95% CI 7.8-51.4) among those who stayed longer than 90 days. Table 2 shows the summary statistics of the intervals and the result of the survival technique analysis. The number of personnel visited during the primary care interval ranged from zero to 4 (median and mode 1, IQR 1-2). The FHP was a general practitioner in the majority of respondents (171 out of 235 (72.8%)) (Figure 1).

Only a few patients reported prior knowledge of breast cancer treatment and outcome, as shown in Table 3. The overall rate of an incorrect directive from the FHP was 26.3 (95% CI 20.5-32.8). More FHP who were non-doctors offered incorrect advice (39.2%) compared to those who were doctors (22.1%) [risk difference 17.1 (95%CI 2.0-31.9) and OR 2.3 (95% CI 1.2-4.4] (Table 4). The rate of long primary care interval was higher among those who received incorrect advice 81% (44 of 54) compared to 67% (100 of 148) among those who received the correct advice (OR 2.1 (95%CI 1.0-4.6)). Symptom misinterpretation and misdiagnosis were the most frequent reasons for prolongation of the primary care interval as shown in Table 3

Exploratory hierarchical agglomerative clustering found four homogenous clusters relatively early. Hence, K-means partitioning optimized 4-cluster solution after sorting in ascending order of unique numbers. Three of the clusters were late presenters (clusters 1 to 3), and one was early presenters (cluster 4).

Cluster 1 patients were homogeneously late presenters, and they were predominantly older than 40 years. They visited only one personnel member before arriving in the specialist clinic, and the FHP correctly advised 92% of them. The main reason for the long primary care interval among cluster 1 patients was the distance to the specialist clinic. Cluster 1 represented patients who are rural dwellers or those residing in areas remote from specialist clinics.

Cluster 2 was the largest. These patients were predominantly younger than 40 years, 95% of them received the correct advice, and most of them visited only one personnel member. Nonetheless, most of them stayed long in the primary care interval. Cluster 2 patients were the most educated and the most knowledgeable about breast cancer treatment and outcome. The majority of patients in cluster 2 made informed decision to delay because of fear of treatment—most probably mastectomy. Cluster 2 represented patients who were not prepared to accept treatment because of their age and need for social acceptability.

Cluster 3 was the smallest in number in this study. The significant factor in this cluster was misdiagnosis and misinformation by the FHP. Clearly, in this cluster personnel error was enormous—an alarming 96% received incorrect advice from their FHP. Although, as in other clusters, the majority of the FHP were doctors, a relatively higher proportion were non-doctors compared to other clusters, and patients in this cluster were the least knowledgeable about breast cancer. More patients in cluster 3 experienced stage migration during the primary care interval compared to other clusters. Cluster 3 represented patients with limited knowledge about breast cancer who rely heavily on the decision of their trusted healthcare provider.

The probability of healthcare provider error was higher among cluster 3, where a relatively higher proportion of FHP were non-doctors. Also, demand for investigation and upward hierarchical referral were rare among cluster 3 patients, and there were more decisions to excise the lesion. This cluster represented situations where the FHP initiated treatment based on incorrect clinical diagnosis without a triple assessment.

Cluster 4 was favorable. Cluster 4 patients were knowledgeable and prepared to accept treatment. Age and proportion of single-unmarried were factors that differentiated cluster 4 (prepared to receive the treatment) from cluster 2 (unprepared to receive the treatment). Cluster 4 patients were predominantly older than 40 years, while those of cluster 2 were predominantly younger. Also, cluster 2 included more single-unmarried women compared to cluster 4.

**Figure 2:**
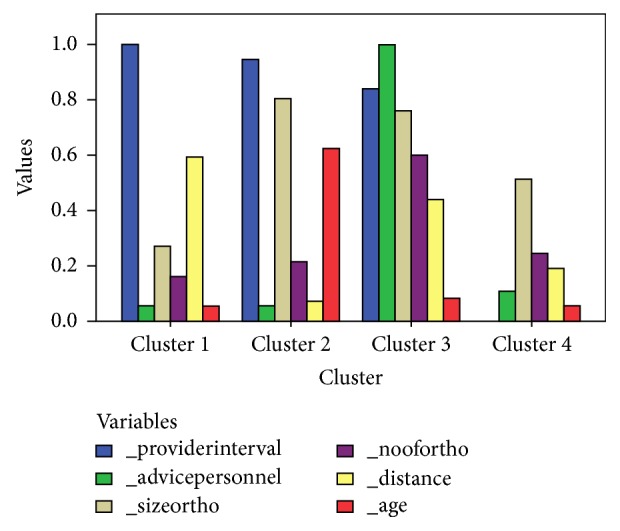
Comparison of proportions of clustering variables. Key. Provider interval: height of bar represents the proportion of patients who stayed longer than 30 days in provider interval; advice personnel: height of bar represents the proportion of patients who received incorrect advice; noofortho: height of the bar represents the proportion of patients who visited more than one personnel member; distance: height of the bar represents the proportion of patients who lived driving distance >60min; sizeortho: height of the bar represents the proportion of patients who had tumors smaller than 3cm; age: height of the bar represents the proportion of patients below 40 years.

Table 3 shows the cluster characteristics, and Figure 2 compares the proportions of optimized variables.

## 4. Discussion

In this research, focused primarily on the impact of the primary care on the risk of breast cancer progression in a black African population, we found the following. (1) long primary care interval was associated with an increased risk of stage migration. (2) The risk of stage migration increased with prolongation of primary care interval. (3) The risk of stage migration in the primary care interval was higher than the risk of stage migration in the patient interval. (4) Four naturally occurring clusters of patient experience and journey were present in the primary care interval.

The overall interval from symptom detection to arrival in the specialist clinic was long for most patients—only a third of the patients arrived at the specialist clinic within 90 days of symptom detection. Also, the mean tumor size increased along the continuum from detection to arrival in a specialist clinic. The average increase in tumor size during the primary care interval was multiple times that of the patient interval. These findings agreed with the trend reported in Nigeria showing an increasing contribution of primary care interval to breast cancer progression and they reflected the positive impact of awareness campaigns and other efforts aimed at shortening the patient interval [19–21].

Studies conducted in Nigeria and other developing countries have suggested the risk of stage migration and worsening of physical characteristics of breast cancer during the patient or primary care interval. None of the studies explicitly reported the magnitude of the risk [19, 21, 25]. Ayoade et al. [19] and Ezeome et al. [21] in Nigeria reported a higher proportion of patients with a late stage in the interval between contact with the FHP and arrival in tertiary institutions. Similarly, Unger-Saldana et al. [6] in Mexico, North America, reported longer stay in the provider interval compared to the patient interval, and they correlated higher proportion of advanced disease with longer intervals. In this study, the risk of stage migration during the primary care interval was more than twice the risk during the patient interval, and the majority of patients who stayed longer than 90 days in the primary care interval experienced stage migration. This study extends the literature in this area of research by providing figures against which interventions can be planned and measured.

Researchers intuitively consider a 2-cluster solution in describing the journey of breast cancer patients in LMICs: the early and the late presenters. The lower level solution found in this research by clustering analysis is a different perspective from which we may gain deeper insights. Joffe et al. [26] used cluster analysis for a different purpose among breast cancer patients in South Africa. In their research, they used cluster analysis to find the sociodemographic subgroups in the time interval to the presentation of breast cancer, but it was not evident in their results how the sociodemographic clustered with the interval. The clustering analysis in this study showed that the late presenters were not a homogenous group.

The clusters found within the general pool of respondents who had long primary care interval in this study were the impact of distance (cluster 1), deliberate delayer (cluster 2), and impact of personnel error (cluster 3). Cluster 2 situation was particularly worrying because they were deliberate delayers. Their age, social aspiration or responsibility, prior experience, and fear of mutilation influenced their decision. Ayoade et al. [19] in Southwestern Nigeria also identified a similarly large pool of deliberate delayers. It is unlikely that simple campaign strategies will help this cluster. The deliberate delayers constitute the subgroup for which the medical community in LMICs must find acceptable and attractive treatment options. Just as patients own the responsibility to present early upon recognition of changes in their breast, the healthcare system owns the responsibility to make appropriate management recommendations [27] and perhaps research to offer attractive treatment options. Specialists and other medical practitioners must understand that healthcare is a service, and when alternatives are available and acceptable in the national policies, patients will prefer less painful choices until they have no choice.

Another finding that was worrying was the high rate of long primary care interval among patients who received the correct directive—60% of upward referrals and 72% of appropriately investigated patients still stayed longer than 30 days in the primary care interval. It is possible that deliberate delaying explains some of the long primary care intervals after appropriate FHP directives, but this area still requires meticulous research. The preintervention Danish report showed a similar trend where cancer patients investigated by general practitioners experienced long waiting time [2]. Strengthening the referral system, tightening oversight monitoring functions, and adopting similar policies used by the Danish system [2] such as specifying maximum waiting time for referral, investigation, and consultation and describing and enforcing fast track referral and treatment systems may assist.

The most frequently perceived reasons for long primary care interval in this study were symptom misinterpretation, misdiagnosis, and systems related factors. Compared to other commonly reported factors such as age, socioeconomic factors, and marital status, the impact of distance to healthcare center and misinterpretation of symptoms are two consistent factors demonstrated in other studies [17, 25, 28]. Symptom misinterpretation and systems related factors were the most common reason for extended intervals in Kenya in a report by Otieno et al. [29]. In a report by Pace et al. in Rwanda, the authors associated longer systems interval with a higher number of visits to other centers before arriving in the referral center [25] or even the specialist center in South Africa [26]. Also, in a study by Moodley in South Africa, symptom misinterpretation was common reason for the long diagnostic intervals [30]. In this study, the incidence of symptom misinterpretation was higher among the younger patients who were also the most knowledgeable cluster. These patients likely wanted to wish the symptoms away because of fear of diagnosis and treatment, which is already a common theme for late presentation among African women [31].

For the first time in Nigeria, this study quantified the direct impact of systems related delays on late presentation, and it also quantified the impact of unguided pluralistic healthcare services on late presentations as was shown in cluster 3. The findings in this survey hold profound implication for strategizing. The longer primary care interval and a higher risk of stage migration in the primary care interval suggest that the patient level interventions are yielding good results. While we should sustain them, we need to divert more resources to provider and systems level intervention through the provision of diagnostic facilities, access to care, transportation, and navigation, education of personnel, and enforcing referral protocols. Currently, most interventions focus on women and the general populace, and we pay little or no attention to providers and systems factors.

The case fatality rate of 60% known to the patients in this study is a significant problem. LMICs need to find means of disrupting the perpetual gloomy outcome of breast cancer. Each time a patient succumbs to breast cancer, especially without delayed presentation to orthodox care or after mastectomy, the surviving friends and family register it as “doom prophesy fulfilled,” and many are likely to join the skeptics who believe that breast cancer is uniformly fatal if treated by orthodox medicine. LMICs must take deliberate steps to increase the number of survivors so that survivors can overshadow case fatalities.

This report is one of the rare articles on primary care interval and factors influencing delay among breast cancer patients in Africa and it is the first to quantify the impact of primary care delay on the risk of disease progression in a black African population. The subjective partitioning done in this research showed a new perspective which may offer opportunities for intervention. Subsequent studies should explore how the different clusters of patients can be recognized and how interventions can be tailored to meet their needs.

Although measures were taken to reduce recall bias in the study design, this survey was still limited in that triangulation with the primary care records was impossible because of poor record keeping. Triangulating may have helped in eliminating the recall bias in the time interval and the estimation of the tumor size progression. However, other attempts to reduce the recall bias included interviewing respondents within 4 weeks of arrival in a specialist clinic. An attempt was made to convert any bias in the tumor size estimate to systematic bias by using the patients' estimate at points, even for the size at the specialist clinic. Additionally, we compared the size reported by the respondent with the size physically measured and excluded inconsistent responses.

## 5. Conclusion

In this research, delay in the primary care interval was associated with a higher risk of stage migration among breast cancer patients, and the risk of stage migration increased with the prolongation of the primary care interval. The most common reasons for long primary care interval were system factors and symptom misinterpretation.

## Figures and Tables

**Table 1:** Sociodemographics and distribution of the first symptom: The modal age range was the 5th decade. The majority of the patients were married. Level of education was tertiary or secondary in the majority. Breast lump was the first symptom noticed by the majority.

Age distribution (years)	n(%)
30 and below	18(7.6)
31-40	51(21)
41-50	74(31.2)
51-60	46(19.4)
61-70	24(10.1)
71 and above	24(10.1)

Age statistics	
mean	48.35±15.9
median	48
IQR	40-58

Marital status	n(%)
married	167(70.5)
single	11(4.6)
divorced or separated	4(1.7)
widow	22(9.3)
unspecified	33(13.9)

Level of education	n(%)
tertiary	91(38.3)
secondary	78(33)
primary	30(12.7)
none	38(16)

First symptom detected	n(%)
breast lump	169(71.3)
pain and itching	18(7.5)
discoloration and sore	3(1.3).
Axillary mass	2(1.0)
nipple discharge	2(1.0)
unspecified	43(18.1)

**Table 2:** Distribution of interval lengths and risk of tumor size migration: The mean and median of the primary care interval were longer than the patient interval. Risk of stage migration increased as the patient stayed longer in the primary care interval.

	Patient interval	Primary care interval	Detection to specialist Interval
Mean (days)	68±172	266 ±315	335 ±413
Median (days)	21	106	195
Range (days)	1-2190	1-2176	1-2190
IQR (days)	7-90	18-356	60-365

Time elapsed (days) vs. the proportion of patients in the interval			
Duration (days)	Patient interval	Primary care interval	Detection to specialist
1-30	145(62.7	67(30.7)	35(15.5)
31-90	42(18.2)	39(17.9)	41(18.1
91- 180	27(11.7)	26(11.9)	37(16.4)
>180	17(7.4)	86(39.5)	113(50)
Tumor size (cm)			
mean	2.8±1.2	3.4±1.2	6.0±4.3
Median (IQR)	3(2-4)	4(2-4)	5(4-8)
range	1-5	1-5	1-24

Tumor stage	At Detection (%)	At FHP contract (%)	At specialist clinic (%)
T1	116 (49.3)	68 (29.5)	32 (15.3)
T2	119 (50.7)	169 (70.5)	100 (42.4)
T3			104 (42.3)

Time elapsed in primary care interval	Number of migration cases (number of patients present at the beginning of period)	Risk of migration during the period (%)	95% CI for risk of migration
30 days	5 (178)	2.8	1.0-6.4
31-90	13 (156)	8.3	4.5-13.8
>90 days	69 (122)	56.6	47.3-65.5

**Table 3:** Classes of advice and errors of the First Healthcare Provider (FHP): The most frequent erroneous advice was attempts to treat by excision or antibiotics. The common reasons for long interval despite correct advice were systems related and symptom misinterpretation. The most frequent directive from the FHP was an upward referral or to investigate.

Distribution of correct advice (n)	Distribution of erroneous advice (n)
Upward hierarchical referral (91)	Antibiotics (26)
Investigating (FNAC, USS, mammo) (65)	Removing (26)
	Observing (5)
	seeking native (1)
Reason for long delay despite correct advice (n)	Reasons for delay in case of incorrect advice (n)
Systems related	Systems related
Awaiting results (7)	Awaiting investigations (2)
Conflicting results (1)	Dislike for tertiary institution (1)
Strike (5)	Strike (1)
Difficult navigation (1)	

Symptom misinterpretation (by the patient)	Symptom misinterpretation
no pain (1)	Pregnancy (1)
Thought benign (2)	
Thought will disappear (10)	

Fear related	
Fear of mastectomy (3)	
Fear of diagnosis (5)	
Fear of biopsy (1)	

Misdiagnosis	Misdiagnosis and mistreatment
Told benign (3)	Told benign (4)
Reassured (1)	Using antibiotics (3)
Not referred (3)	Reassured (1)
	Not referred (1)

Socioeconomic and cultural	Socioeconomic and cultural
Financial issues (2)	Financial issues (6)
Social responsibility (2)	Spiritual solution (1)
Using herbs (1)	Using herbs (3)

Distribution of directives	Length of primary care interval: ≤ 30 days/ > 30 days
Upward referral	30 / 45
Investigating	18 /46
Excising	4/24
Antibiotics/medications	5/19
Reassuring/observing/seeing native	4/8

**Table 4:** Cluster characteristics: There were three subgroups with long primary care intervals (clusters 1-3) and one with short primary care interval (cluster 4). The majority of the patients were aware of breast cancer in all the clusters. The largest cluster was the deliberate delayers.

	Cluster 1	Cluster 2	Cluster 3	Cluster 4
	(n=36)	(n=56)	(n=26)	(n=39)
Clustering variables				
Primary care interval	>30 days	>30 days	>30 days	<30 days
Directive	Correct	Correct	Incorrect	Correct
Tumor size at FHP contact	>3cm	<3cm	<3cm	<3cm
Number of personnel visited	one	one	>one	one
Driving distance (min) to specialist	>60	<60	<60	<60
Age	>40	<40	>40	>40
Cluster label	Correct advice and late (older)	Correct advice and late (younger)	Erroneous advice and late	Correct advice and early
Knowledge distribution across clusters				
Aware of breast cancer (%)	72	87	68	79
Knowing treatment (mastectomy)	6	13	3	8
Knowing patient	7	12	7	10
Knowing case fatality	4	6	2	6
Knowing case survival	2	3	3	4

Perceived reason for the delay				
misdiagnosis		1	6	3
Symptom mis-interpretation (by patient)	4	6	1	2
Systems (awaiting result, navigation, strike)	3	8	2	1
fear of mastectomy	3	2	0	1
financial and social issues	4	5	3	1
spiritual and native healing	1		1	

Type of First Healthcare Provider				
doctor	31	41	15	33
others (nurse, CHEW, chemist, pharmacist)	5	15	11	4
Distribution of FHP directives				
upward referral	17	31	0	24
investigating	17	22	1	9
excising	1	3	9	4
antibiotics/medications	2	0	16	0
Age distribution (years)				
Range	31-80	26-78	32-75	34-85
Mean/median/mode	56/56/43	42/40/40	53/52/42	56/52/47
IQR	47-65	34-48	46-62	47-65
Marital status				
married	25	45	17	28
single		5		1
Separate/divorced/widow	3	4	7	5
Educational status > 6 years (%)	67	76	65	59
Experienced stage migration (%)	53	50	58	22
Deduction	Impact of distance and lack of resources (systems)	Deliberate delayers (socially unprepared to accept treatment The knowledgeable and independent decision maker	Impact personnel error Not knowledgeable and dependent on personnel for decision	Prepared to accept treatment

## Data Availability

The data used to support the findings of this study are available from the corresponding author upon request.
